# An Effective and Efficient Dynamic eMBMS Multicast Grouping Scheduling Algorithm in MBSFNs for Public Safety Scenarios

**DOI:** 10.1109/access.2020.3000251

**Published:** 2020

**Authors:** SIYUAN FENG, CHUNMEI LIU, CHEN SHEN, HYEONG-AH CHOI, RICHARD A. ROUIL

**Affiliations:** 1 Department of Computer Science, The George Washington University, Washington, DC 20052, USA; 2 Wireless Networks Division, National Institute of Standards and Technology, Gaithersburg, MD 80305, USA; 3 Department of Physics, Georgetown University, Washington, DC 20007, USA

**Keywords:** eMBMS, LTE, MBSFN, mission critical, multicast, public safety, RAN, scheduling

## Abstract

The LTE (Long-Term Evolution) eMBMS (evolved Multimedia Broadcast Multicast Service) technology employed in MBSFNs (Multicast Broadcast Single Frequency Networks) has been shown to be capable of considerably increasing the capacity of serving public safety users under group communication scenarios. However, due to its own limitations, a more fine-tuned scheduling approach is needed in order to fully utilize the strengths of eMBMS multicast. In this work, we first identify and analyze several overlooked challenges for scheduling multicast traffic in MBSFNs. Then we develop an effective and efficient dynamic scheduling algorithm for eMBMS multicast in time and frequency varying channel. The proposed algorithm leverages the advantages of both multicast and unicast schemes via user grouping; and through extensive simulations, is shown to be capable of significantly enhancing the mission critical performances under both best effort and guaranteed bit rate delivery models. We also show the resiliency of our proposed algorithm by applying it onto various network and user deployment scenarios. Our consistent results further prove that LTE eMBMS in MBSFN is a key solution in overcoming limitations in near future public safety networks.

## I. INTRODUCTION

In public safety scenarios, significant amount of massive mission-critical group traffic is expected among First Responders (FRs) [1]. Due to this fact, the LTE (Long-Term Evolution) eMBMS (evolved Multimedia Broadcast Multicast Service) technology is considered to be a prominent solution over the point-to-point unicast scheme for enhancing the capacity, coverage, and resilience of Public Safety Networks (PSNs). Key research labs, such as Samsung,^1^ recently proposed to overcome PSN limitations through eMBMS [2].

The utmost significant deployment environment for eMBMS multicast deliveries is MBSFNs (Multicast Broadcast Single Frequency Networks), where multiple tightly synchronized and coordinated eNBs (evolved-NodeBs) in the MBSFN area are to broadcast the exact same content at the exact same time over shared resources. In this way, the traditionally destructive Inter-Cell Interference (ICI) becomes constructive Multi-path factors for UEs (user equipment) within the MBSFN area (denoted as MBSFN UEs). Consequently, UEs who can successfully combine these eMBMS multicast signals will benefit from the diversity, and thus will attain higher SINRs (Signal-to-Interference-and-Noise-Ratios), i.e., the MBSFN gain, especially those at sector/cell edges.

**FIGURE 1.**
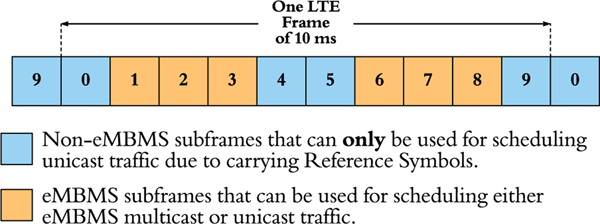
LTE Downlink eMBMS subframe structure: eMBMS subframes (1, 2, 3, 6, 7, 8) and non-eMBMS subframes (0, 4, 5, 9) in an LTE Frame, where each square block occupies 1 ms (horizontally).

As shown in our previous work [3], for the MBSFN setup illustrated in Figure 3, UEs’ achieved throughput were substantially increased when multicast was deployed, despite that eMBMS^2^ traffic can only utilize up to 60 % of the resources in each LTE Frame [4] (refer to Figure 1).

However, in that work, we’ve only compared the following two baseline scheduling approaches: (Here, efficiency is referring to the efficiency of channel utilization, or spectral efficiency.)

- **All Unicast**, where all UEs were up to be scheduled for unicast traffic via Proportional Fairness Scheduling (PFS) algorithm; which was obviously not efficient in multi-user cases due to the ineptitude of unicast traffic for *i*) sharing radio resources among UEs on duplicative data and *ii*) attaining stronger SINRs during transmissions, especially for remote UEs; or
- **All Multicast**, where all UEs were participating in eMBMS multicast traffic; which on the other hand, was neither sufficiently efficient since *i*) the Transport Block (TB) employed for the entire MBSFN had to accommodate UEs with the worst available Modulation and Coding Scheme (MCS), and *ii*) UEs with MIMO (Multiple-Input Multiple-Output) capabilities wouldn’t be able to utilize this advantage at all.

Therefore, in this work, we aim to develop a more fine-tuned dynamic multicast scheduling algorithm that better leverages the advantages of both eMBMS multicast and unicast traffic for UEs in MBSFNs.

Also, in [3], as well as in majority of existing literature, the performance of a proposed algorithm was only measured through its *capacity*, i.e., the best effort throughput achievable by the network UEs. However, since the best effort scheduling model lacks QoS (Quality-of-Service)-awareness, individual UEs’ throughput values could differ drastically, even though fairness is often considered. Whereas in the case of Mission-Critical Communications (MCC), since it is desired to ensure as many FR UEs as possible to receive their demanded service, the network needs to guarantee that all of the UEs can at least achieve some certain minimum bit rate requirement for the service, known as the Guaranteed Bit Rate (GBR) of the application. Hence, besides the system capacity, we will also investigate the *coverage* performance of the network, i.e., the percentage of UEs in the network that can reach the service GBR.

In summary, **we wish to develop an eMBMS multicast scheduling algorithm that, on one hand, aims to maximize the capacity for UEs in an MBSFN; and on the other hand, guarantees certain coverage for these UEs.** The proposed algorithm will decide which of the UEs are going to be grouped together for multicast traffic based on choosing a bottleneck channel quality value, such that the throughput within each eNB sector is maximized, where the grouping is periodically re-iterated in response to UEs’ varying channel qualities. We claim that our proposed algorithm is: *i)*

1. **dynamic**, because it can effectively handle UEs’ frequency selective and time varying channel qualities; and
2. **effective and resilient** that it will, almost always, significantly out-perform baseline algorithms under various network and UE deployment scenarios, and usually regardless of the application; as well as
3. **efficient and practical**, since it requires a low computation complexity, and is ready to be directly adopted for standard-compliant implementations under current or envisioned near future PSNs.

The rest of this work is organized as follows, in Section II, we review several related works and discuss how our proposed approach is different from theirs. In Section III, we further elaborate some of the challenges that exist in the multicast scheduling problem, which were not dealt with by the existing literature, but that we aim to tackle in this work through various novel heuristic approaches. To the best of our knowledge, this is the first multicast scheduling work to explicitly address and handle these mentioned issues. In Section IV, we present our proposed dynamic multicast grouping scheduling (**DMGS**) algorithm with detailed explanations and a brief discuss on its time complexity. In Section V, we present extensive simulation results of the proposed algorithm, and discuss its performances and resiliency. Lastly, we conclude our paper and briefly talk about future work and technical trends of eMBMS in Section VI.

## II. RELATED WORK

One common issue presented in many existing eMBMS scheduling works was that, an MBSFN UE was associated with the same unicast and multicast MCS value. However, due to the MBSFN gain factor, this is conceptually not true; and in fact, two separate sets of channel quality data should be considered for each of the UEs. Consequently, these works failed to identify this extra variant dimension in their formulated problems and proposed solutions. In this work, we will effectively utilize both in our proposed solution.

Meanwhile, various works aimed to enhance eMBMS performances through conducting network optimizations. For example, in [5] and [6], the authors proposed to use different eNB clustering techniques in the MBSFN to improve the system performance with multiple contents in demand. In [7], the authors proposed a more efficient feedback mechanism for eMBMS UEs in order to improve their QoS performance.

In [8], Alexious *et al.* proposed four adaptive MCS selection algorithms. However, except for the bottom-up algorithm which selects the minimum MCS capable for all UEs in the MBSFN, essentially the all multicast scheduling scheme mentioned earlier, the others cannot guarantee all the UEs to successfully decode the transmitted multicast TBs.

In [9], Fuente *et al.* proposed a QoS-aware joint multicast/unicast scheduling algorithm that was also based on choosing the multicast bottleneck value, such that the network throughput is maximized. However, they discounted the distinctive functionalities of different network entities; namely, multicast traffic is governed by the MCE (MBMS Coordination Entity) for the whole MBSFN, whereas unicast traffic is handled locally by the eNB sectors. Hence, to optimally maximize the network throughput, the eNBs and the MCE will need to constantly exchange information, which will lead to extra overheads. To resolve this issue, this work, instead, focuses on maximizing the sector throughput, which we will further explain in detail in Section IV.

In [10], Chen *et al.* proposed an eMBMS multicast subgroup partitioning algorithm among pre-determined multicast UEs in an MBSFN, such that the user experience for UEs with good signal qualities will not suffer by being grouped together with those with poor signal qualities. In [11], Ghandri *et al.* pointed out that the former was not compliant with the standard regarding the granularity of resources allocated to eMBMS traffic; and they proposed a standard compliant scheduling algorithm based on similar idea. However, we have two major disagreements with the idea of these works.

§ Firstly, both proposals assumed that the multicast group was pre-determined, since the unicast UEs were demanding contents other than the one offered by the multicast service. However, due to this fixed pre-arrangement, the MIMO capabilities of those in the multicast group were not taken into consideration in the decision making procedures at all.

§ Secondly, they aimed to enhance the system performance by trying to divide the multicast UEs into subgroups. However, this approach is not applicable for PSN UEs; since in their case, UEs with non-ideal channel qualities might never be able to receive the same content as those with better channel qualities, and would hence fail to receive the mission-critical message.

Therefore, it is reasonable for us to argue that, for users demanding a unified content, instead of further dividing them into multiple multicast subgroups, we simply **form one multicast group**, and **exclude some of the UEs from receiving eMBMS deliveries for some specific time periods**, so that they will not drag down the overall efficiencies for multicast traffic; and, **as remedies, try providing unicast deliveries to these UEs** based on PFS metrics, where now their MIMO capability is going to be utilized. In this way, **all UEs can have the opportunity to receive the same content**, despite that some may need longer times than others.

One last issue presented in [10] and [11] was that, their solutions were based on the notion of UEs’ “effective MCS” values, which, in their work, were considered as fixed values for each UE throughout time and across the spectrum. However, in a communication channel, the channel qualities, for both multicast and unicast traffic, are in fact frequency selective and time varying. Thus, both works were lacking on how to deal with these extra dimensions of variances in their solutions. We further discuss on how we propose to resolve this issue in the next section.

## III. PROBLEM SCENARIO AND RELATED CHALLENGES

### A. USERS’ VARYING CHANNEL QUALITIES

As mentioned above, the channel qualities for both unicast and multicast traffic are frequency selective and time varying. Hence, even if perfect channel knowledge is assumed, the optimal solution for the joint scheduling across some transmission time period is likely to take a rather high order to solve. Thus, in order to perform real-time dynamic scheduling, we will utilize the following described approaches to approximate UEs’ channel qualities. Also, in this work, instead of focusing on UEs’ MCS values, we consider their CQIs (Channel Quality Indicators) and RIs (Rank Indicators).

#### 1) FREQUENCY SELECTIVITY

Since LTE Downlink (DL) transmissions would experience frequency selective fading, the channel qualities experienced by a UE would vary on each of the Physical Resource Blocks (PRBs).

Luckily, for multicast deliveries, since a single TB is to be formed for each eMBMS subframe [12], hence before the MCE chooses the coding scheme to be applied for the MBSFN, the eNBs will first utilize the Mutual Information Effective SINR Mapping (MIESM) [13] to obtain a single effective TB CQI for each of its attached UEs. Thus, reduces one variant dimension for the scheduling problem.

On the other hand, for unicast deliveries, since we will be utilizing PFS, which takes UEs’ real-time CQI values to determine *i*) the UEs that are going be scheduled for current TTI (Transmission Time Interval), as well as *ii*) the PRBs that are going be allocated for each of them, we will keep UEs’ unicast CQI structure as it is.

Table 1 showcases an example of a UEs’ experienced channel qualities at each TTI. In the table, the multicast CQI for each subframe is presented as the post-MIESM effective TB CQI; where since TTI 35 is an non-eMBMS subframe (refer to Figure 1), the multicast CQI at that subframe is zero. On the other hand, for unicast, if the UE is capable of decoding more than one transmitted layers at certain TTI, then there will be two codewords available for the UE, with each codeword associated with one CQI value; and if not, then the second codeword’s CQI is going to be unavailable.

#### 2) TIME VARIANCE

Meanwhile, due to fast fading attenuation, non-stationary UEs’ channel qualities are time variant. Therefore, to ensure the effectiveness of the grouping result after some time period, re-scheduling is needed.

As pointed out by [11], works such as [9] and [14] considered multicast scheduling at a per-TTI-level. Although this would resolve the time variant issue with real-time accuracy, huge amount of signaling overheads would be introduced into the communications. Hence, Ghandri *et al.* suggested to perform multicast scheduling once every *T*-subframe period. However, there was no mention on how to choose the proper value of *T* for it to be of both effective and efficient, as well as having any valid underlying significance.

In this work, we propose to utilize the notion of **Channel Coherence Time** for first sampling UEs’ channel qualities, and then decide the multicast grouping based on these sampled values for the upcoming coherence time scheduling window. We claim that this approach will not only reduce the scheduling complexity and overhead, but also ensure certain accuracy in the approximations, which will eventually lead to favorable performances. We later validate this claim through our extensive simulations presented in Section V; and further elaborate our coherence time sampling approach below.

### B. CHANNEL COHERENCE TIME SAMPLING

The channel coherence time $T_{c}^{*}$ over a wireless communication channel, specified in Eqn. (1), is defined as *the period of time where the channel is considered to be static over fast fading caused by Doppler effect* [15]; where *f*_*m*_ is defined as the maximum amount of Doppler spread of the channel.

**Channel Coherence Time:**

(1) $$T_{c}^{*}=\sqrt{\frac{9}{16\pi }}\cdot \frac{1000}{f_{m}}\approx \frac{423}{f_{m}}=423/\left(\frac{v}{c}\cdot f_{c}\right)\;\text{ms}$$

where *c* is the speed of light, and *v* is the maximum object moving speed, and *f*_*c*_ is the central frequency of the transmission spectrum, where for PSNs with Band 14 DL, *f*_*c*_ = 763 × 10^6^ Hz.

In Table 2, the coherence time for each of the commonly employed International Telecommunication Union (ITU) channel mobility models are listed, where **Ped** refers to pedestrian model and **Veh** refers to vehicular model. In the table, the *T*_*c*_ values are the corresponding coherence time values rounded up to the nearest millisecond; and we utilize *T*_*c*_ as the described coherence time *scheduling window*.

Based on the coherence time concept, within one *T*_*c*_ period, the channel qualities of a UE can be treated as fixed values. Hence, as described above, first at the beginning of each *T*_*c*_ window, every UE’s multicast and unicast channel qualities are to be sampled for that UE for that scheduling window, and these samples are then used for determining which of the UEs are to be included into the multicast group.

Consequently, within one *T*_*c*_ period, a UE is dedicated to receive only one type of traffic, either unicast or multicast. This will hence greatly reduce the signaling overheads introduced by frequent switching of a UEs’ reception schemes; yet would still provide enough dynamics towards UEs’ time varying channel condition through this proper re-scheduling time interval. To the best of our knowledge, this is the first work to utilize this concept to perform UE scheduling.

Our proposed novel coherence time scheduling approach is valid for LTE eMBMS traffic since it concurs with the eMBMS subframe structure specified in [4]. In Figure 1, it can be seen that, within one LTE Frame, subframes 0, 4, 5, and 9 are used to carry the LTE DL Reference Symbols (RSs), hence only subframes 1, 2, 3, 6, 7, and 8 can be used for scheduling multicast transmissions.

More specifically, for example, consider a channel associated with the **Veh-A** model. If we are now at the *t*^th.^ TTI, and this TTI corresponds to the 0^th.^ subframe in an LTE Frame, then at the current TTI, the eNBs are going to receive the channel quality feedbacks that the UEs estimated in the previous TTI, i.e., the (*t*−1)^th.^ TTI. Due to channel coherence time, these just received channel qualities, i.e., the sampled channel qualities in our proposed scheme, can then be treated as static values up to the (*t* + *T*_*c*_ − 1)^th.^ TTI.

**FIGURE 2.**
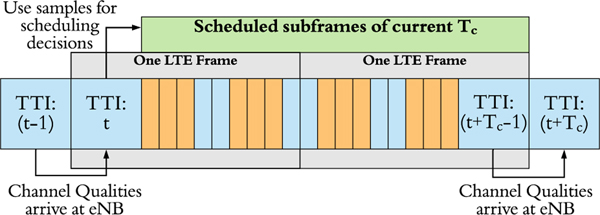
Proposed channel quality sampling and scheduling approach that combines the notion of channel coherence time *T_c_* and the LTE eMBMS subframe structure. Again, the orange and blue blocks represent eMBMS and non-eMBMS subframes, respectively.

Hence, since *T*_*c*_ = 20 ms in this channel, at the *t*^th.^ TTI, we can schedule the UE traffic for the next two LTE Frames using these sampled data without the need of worrying about drastic variations in the channel. As illustrated in Figure 2, for our described example, at TTI *t*, the eNBs will utilize TTI (*t* − 1)’s channel qualities to make the scheduling decisions for the next four sets of eMBMS subframe triplets and three sets of non-eMBMS subframe pairs, plus for the subframe at TTI (*t* + *T*_*c*_ −1). Then, at the (*t* + *T*_*c*_) TTI, the same sampling and scheduling process is going to be conducted again for the next *T*_*c*_ window period.

Note that, since at TTIs (*t* + *k* · *T*_*c*_), for *k* ∈ ℤ*, the grouping arrangements for the scheduling periods are still undecided, the resources in these TTIs can not be utilized by the mission-critical UEs. Later we can see that this impact is overall negligible. However, these resources can then be used for serving other regular UEs in the network through unicast.

### C. eMBMS SUBFRAMES AND MULTICAST TRAFFIC

Since eMBMS subframes are capable of delivering both unicast and multicast traffic, we will need to decide the traffic type for each of these subframes. Unlike the algorithms proposed by [9] and [11] where the number of subframes used for multicast traffic was investigated as variables; in this work, we argue that all the eMBMS subframes should be utilized for multicast traffic, as explained below.

Based on the widely adopted log-utility approach presented in [10], for *k* UEs to be scheduled in an eMBMS subframe *t*, we argue that, the subframe should be used for unicast if and only if the approximated achievable TB size of unicast traffic (with added MIMO component) is greater than that of multicast traffic in that subframe; i.e.,

(2) $$k\cdot \log\left(50{\tilde{c}}_{k}\right)<\sum_{i=1}^{k}\log\left(y_{i}^{t}\cdot {\tilde{d}}_{i}^{t}\cdot r_{i}^{t}\right);$$

where $\tilde{c_{k}}$ is the multicast coding scheme (bits/PRB) among these UEs at subframe *t*, which corresponds to their lowest CQI ${\hat{c}}_{k}$, and 50 is the number of PRBs allocated for eMBMS in Band 14; and ${\tilde{d}}_{i}^{t},r_{i}^{t}$, and $y_{i}^{t}$ are the unicast coding scheme, RI, and the number of assigned PRBs for UE *i* at the current subframe, respectively. Then, if we assume all UEs have similar unicast channel qualities and are, hence, assigned with similar amounts of PRBs in unicast deliveries, we can maximize the right-hand side, and obtain the inequality,

(3) $$k/r_{i}^{t}\cdot \tilde{c_{k}}<{\tilde{d}}_{i}^{t}.$$

However, since in most cases, it is true that $k\gg r_{i}^{t}$; therefore, the unicast CQI needs to be several times of the multicast CQI in order to make Eqn. (3) hold, which empirically, due to the MBSFN gain factor, is rarely true.

Therefore, in our proposed scheduling algorithm, multicast traffic is being **prioritized** over unicast traffic. Specifically,

- for the upcoming *T*_*c*_ window period, all the eMBMS subframes are reserved for scheduling multicast traffic;
- after a bottleneck value is chosen for multicast traffic, all UEs who are capable of decoding the multicast TB with their sampled CQIs, as a by-product, will be included into the multicast group; this will help save resources in the spectrum and hence provides more service margin for under-capable UEs; and lastly
- given a multicast bottleneck, unicast traffic will then be scheduled for under-capable UEs, where now MIMO capability will also affect the scheduling decision.

### D. PROBLEM SCENARIO

In summary, in this work, we consider a PSN scenario where the FR UEs are deployed to an incident scene that is covered by an MBSFN. Furthermore, all the UEs are expecting to receive some unified mission-critical content that has a service GBR of Q b/s, provided by some central authority. Given the network, we aim to enhance the MCC channel performance by dividing the FR users into receiving either multicast or unicast deliveries within each scheduling window of *T*_*c*_ subframes based on choosing a bottleneck CQI for the multicast group. We describe our proposed grouping scheduling algorithm in the following section.

## IV. eMBMS MULTICAST SCHEDULING

The layout of our proposed algorithm is in two parts, namely, Protocol 1: Dynamic Multicast Grouping Scheduling, and Algorithm 2: Sector Multicast Decision. We note that while the multicast CQI selection for the MBSFN is done at the MCE side; the majority of the steps are going to be executed at the MBSFN eNB side, hence can be done concurrently.

### A. SCHEDULING FRAMEWORK AND GROUPING DECISION

In Protocol 1, we present the scheduling framework that defines the functionalities on *i*) differentiating the subframes within the TTIs, and *ii*) providing the corresponding actions associated with these different types of subframes.

#### § Differentiating the Subframes:

- First, if the current TTI index modulo 10 is a member of the set {1, 2, 3, 6, 7, 8}, then it is going to be utilized for transmitting eMBMS signals following the grouping arrangement decided for the current *T*_*c*_ window.
- Next, if the current TTI is not an eMBMS subframe, and the value of the variable *countdown*, i.e., the countdown for re-scheduling, has reached zero, then this TTI is going to be utilized for sampling UEs’ channel qualities and performing the grouping and scheduling for the upcoming scheduling window.
- Lastly, if the current TTI is of neither the above, then it is going to be used for scheduling and transmitting unicast signals for UEs that are not included in the multicast group in the current scheduling window.

Then, for each of the different types of subframes, we define the following associated actions. § **eMBMS Scheduling Subframes**: If the current TTI is a sampling/scheduling TTI, then the MCE will need to decide the bottleneck CQI value *χ* employed for the MBSFN for the current scheduling window. Thus, in each MBSFN sector, we try to maximize the predicted multicast-unicast combined sector throughput, by choosing a multicast CQI bottleneck for that sector based on its UEs’ sampled channel qualities. As presented in Algorithm 2, based on our prioritization of multicast traffic, we perform the following actions:

- First, we assume *c*′ being the multicast CQI for the current sector, and compute the aggregated TB size that can be achieved by all capable UEs within one scheduling window, which is (0.6 · *T*_*c*_) subframes.
- Then, for the remaining UEs in the sector, we apply PFS upon them in the non-eMBMS subframes, and compute the aggregated TB size achieved by these unicast UEs across the scheduling window. Thus, we can combine the unicast and multicast parts to obtain the sector throughput *η*_*c*′_ under current multicast bottleneck.
- Based on the steps described above, we iterate all the possible *c*′ values, and pick the one that would maximize the sector throughput as the chosen multicast CQI bottleneck for current sector. In case there are several such values, we pick the smallest one.

**Protocol 1:** Dynamic Multicast Grouping Scheduling

**Input:** UEs’ channel quality data and the amount of bits already transmitted by them so far in the current second; *T*_*c*_ and Q;
1: Initialize *countdown* value;
2: **for** (each TTI *t*) **do**
3: **if** (*t* is an eMBMS subframe) **then**
4: Update the total TB sizes by Γ for eligible multicast UEs;
5: **else if** (*t* is an sampling/scheduling TTI) **then**
6: **for** (each MBSFN sector *k* ∈ *I*_eNB_, concurrently) **do**
7: Let $\hat{c_{k}}$ ← Algorithm 2, and send $\hat{c_{k}}$ to the MCE;
8: **end for**
9: *countdown* ← *T*_*c*_;
10: [**at the MCE**]: $\chi \leftarrow \min_{k\in I_{\text{eNB}}}\left\{{\hat{c}}_{k}\right\}$, and distribute *χ* back to the MBSFN eNBs;
11: Let UEs who currently have sampled multicast CQIs no less than *χ* be in the multicast group *M* for current scheduling window;
12: Given *χ*, compute the eMBMS TB size, г, achievable by a single eMBMS subframe;
13: **else**
14: **for** (each MBSFN sector, concurrently) **do**
15: Apply PFS using real-time channel qualities onto UEs who are not in the multicast group;
16: **end for**
17: Update TB sizes of unicast UEs;
18: **end if**
19: Update transmission list;
20: *countdown* ← (*countdown* − 1);
21: **end for**

Hence, for each MBSFN sector *k*, we can concurrently pick the multicast bottleneck $\hat{c_{k}}$ that maximizes the throughput for this sector.

Then, the sectors will have their picked $\hat{c_{k}}$ values sent to the MCE, such that the MCE can then choose an appropriate value for the MBSFN. Here, a compromise in favor of decision time is going to be made by having the MCE simply picks the lowest $\hat{c_{k}}$ value among all the received ones. Otherwise, the MCE will need to perform yet another optimization over all the UEs, which was why the algorithm proposed in [9] would not work properly or efficiently. We denote this agreed MBSFN CQI value as *χ* for the current scheduling window. Lastly, after *χ* is distributed back to the MBSFN eNBs, the eNBs are going to arrange their multicast groups based on this agreed value.

**Algorithm 2:** Sector Multicast Decision

**Input:** current sector UEs’ channel quality data and *T*_*c*_;
**Output:** predicted optimal multicast CQI for this sector.
1: Let R ← Ø;
2: Among current sector UEs, obtain set C containing all the unique sampled multicast CQI values for current scheduling window;
3: **for** (each multicast CQI value *c*′ ∈ C) **do**
4: Given *c*′ being the hypothetical MBSFN CQI, compute the multicast TB size, *γ*, achievable by an single eMBMS subframe for a single UE;
5: Obtain *μ*, the number of UEs who have sampled multicast CQIs no less than *c*′, i.e. the capable UEs;
6: Compute the predicted sector multicast TB size of current scheduling period, *η*_*M*_ ← *μ* · *γ* · (0.6 · *T*_*c*_);
7: Hypothetically, apply PFS in the remaining non-eMBMS subframes onto UEs who have sampled multicast CQIs worse than *c*′;
8: Compute the predicted sector unicast TB size of current scheduling period, *η*_*U*_, as the sum of the TB sizes of all unicast UEs;
9: R ← R ∥ (*c*′*, η*_*c*′_ = *η*_*M*_ + *η*_*U*_);
10: **end for**
11: **return** the smallest multicast CQI *c*′ that results the largest *η*_*c*′_ among all the pairs in R;

#### § Multicast/Unicast Transmission Subframes:

Whereas if the current TTI is for transmitting either multicast or unicast signals, the eNBs will simply need to follow the grouping arrangement decided for the current scheduling window, and we will update the TB sizes for corresponding recipient UEs.

Note that for unicast scheduling, now UEs’ CQIs are adapted to their real-time channels instead of using the fixed sampled values. Whereas, since the multicast traffic will be using the agreed *χ* value based on the channels when samples were taken, and the real-time multicast channels experienced by a UE could actually fluctuate, if at any point the real-time channel quality of a UE falls below the sampled value, it will fail to decode the multicast TB at this instance.

Therefore, we are going to update a UE’s received TB size only if this UE can actual successfully decode the corresponding TB, or “eligible” as we described in the algorithm.

#### § Status Update:

Lastly, before we proceed to the next TTI, to ensure *coverage*, we need to update the transmission status. Specifically, for UEs who would achieve the GBR after successful reception of the latest TBs, we will remove them from the transmission list. In this way, these UEs will no longer be considered for future scheduling procedures; and hence, would provide more opportunity for those who are currently lacking on throughput. In addition, when a new second is reached, the list is refreshed.

We can note that, given our framework, in order to perform best effort scheduling, we can simply set Q to a rather large value, such that none of the UEs can actually achieve such throughput within a one second period.

### B. TIME COMPLEXITY

We claim that our proposed algorithm is efficient, where one of the main reasons is that, as mentioned earlier, most of the computations are done at each of the eNB sectors concurrently. Notably, since PFS imposes a quadratic time complexity each time it is performed, our algorithm can indeed cut considerable amount of computation time when compared with algorithms that deal with all network UEs altogether. Specifically, our proposed algorithm has a time complexity of *O*(*Cn*^2^) at the scheduling TTIs due to the PFS part, where *n* is the number of UEs within each MBSFN eNB sector and *C* is the fixed number of CQI values that are allowed for eMBMS. For unicast transmission TTIs, we will have a similar quadratic time complexity of *O*(*n*^2^). For multicast transmission TTIs, the complexity is *O*(*n*), since we only need to update UEs’ current TB sizes and check if we need to remove them from the transmission list.

If we were to consider all the network UEs, *N* of them, in the optimization, then the time complexity of *O*(*N*^2^) is going to significantly slow down the scheduling process; and might make it impossible for real-time scheduling.

## V. SIMULATED RESULTS

**FIGURE 3.**
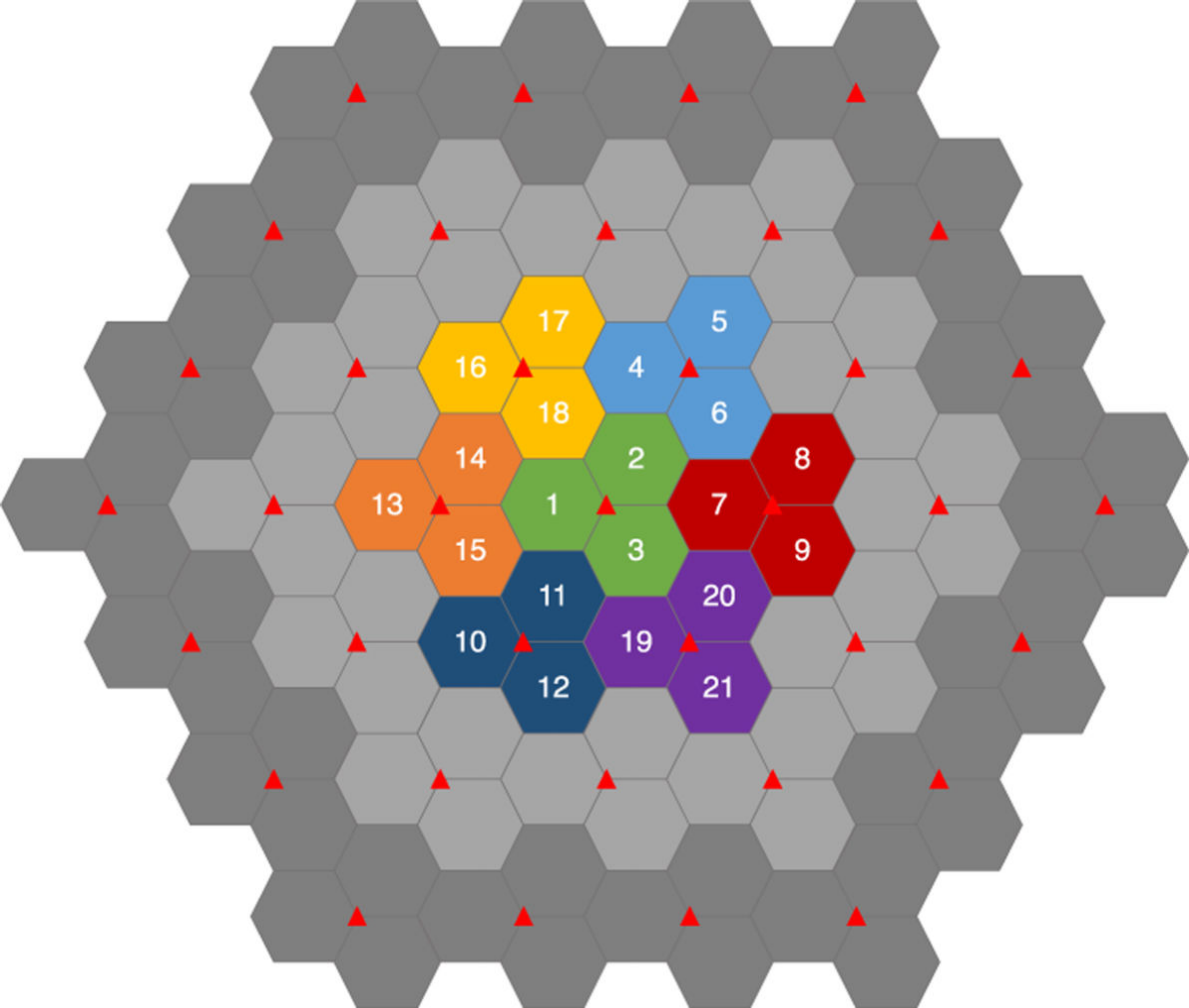
Network layout with cell sector IDs: a network consisting of 37 eNB sites (red triangles). For our baseline scenario, the 7 central sites (with colorful sectors 1–21) form an MBSFN; while all the outer-ring sites (with light and dark grey sectors) are acting as interference sites to the MBSFN.

Finally, in this section, we showcase the performance of our proposed eMBMS scheduling algorithm using simulated channel quality data under various MBSFN deployment scenarios. The details of the investigated MBSFN and UE deployment scenarios are listed in Table 3. In general, we consider a network area that consists of 37 eNB cell sites, and for each of the cell sites, a standard tri-sector layout is considered, as illustrated in Figure 3. Each cell sector uses its own set of 10 MHz physical resources within the Band 14 DL spectrum dedicated to PSNs. The UEs dropped in the MBSFN area are under the **Veh-A** mobility model, and are capable of decoding up to 64-QAM signals [16], with 8 × 4 MIMO capability under Transmission Mode 9.

We have fully and truthfully implemented the proposed **DMGS** algorithm, and hence obtained the following performance results for the various tested public safety scenarios. For the channel quality simulations, we employ the Vienna LTE-Advanced DL System-Level Simulator [13] and its eMBMS module developed by Liu *et al.* [3] to obtain MBSFN UEs’ TTI-level unicast and multicast channel qualities.

### A. PERFORMANCE OF PROPOSED ALGORITHMS

Below, we compare the performance results of our proposed **Dynamic Multicast Grouping Scheduling (DMGS)** algorithm over the deployment scenarios against the performance of the following baseline algorithms,

- **All Unicast Scheduling (AUS)**: PFS where all UEs are up to be scheduled through unicast at each subframe;
- **All Multicast Scheduling (AMS)**: eMBMS scheduling where all UEs are served through multicast at every eMBMS subframe with the current worst multicast CQI;
- **All Multicast Scheduling with Sampling (AMSS)**: eMBMS scheduling where all UEs are served through multicast at every eMBMS subframe with proposed coherence time CQI sampling scheme, i.e., the CQI being applied for the MBSFN within each *T*_*c*_ = 20 ms window is the lowest value sampled from the beginning of the scheduling windows.

Below, we present the performance results and our in-depth analyses.

### B. SCENARIO I. BASELINE SETTINGS

This family of scenarios is aimed at comparing the algorithms in a generic setting, where we consider a quite large MBSFN area and the FR UEs within are uniformly randomly distributed. We test two cases for this scenario, where in **Sc I.a.**, each MBSFN sector contains 5 UEs, and in **Sc I.b.**, each MBSFN sector contains 10 UEs. In these scenarios, due to the amount of sites contributing to constructive ICI, we are expecting that the UEs within the central 21 eNB sectors are to experience significant amount of MBSFN gains.

**FIGURE 4.**
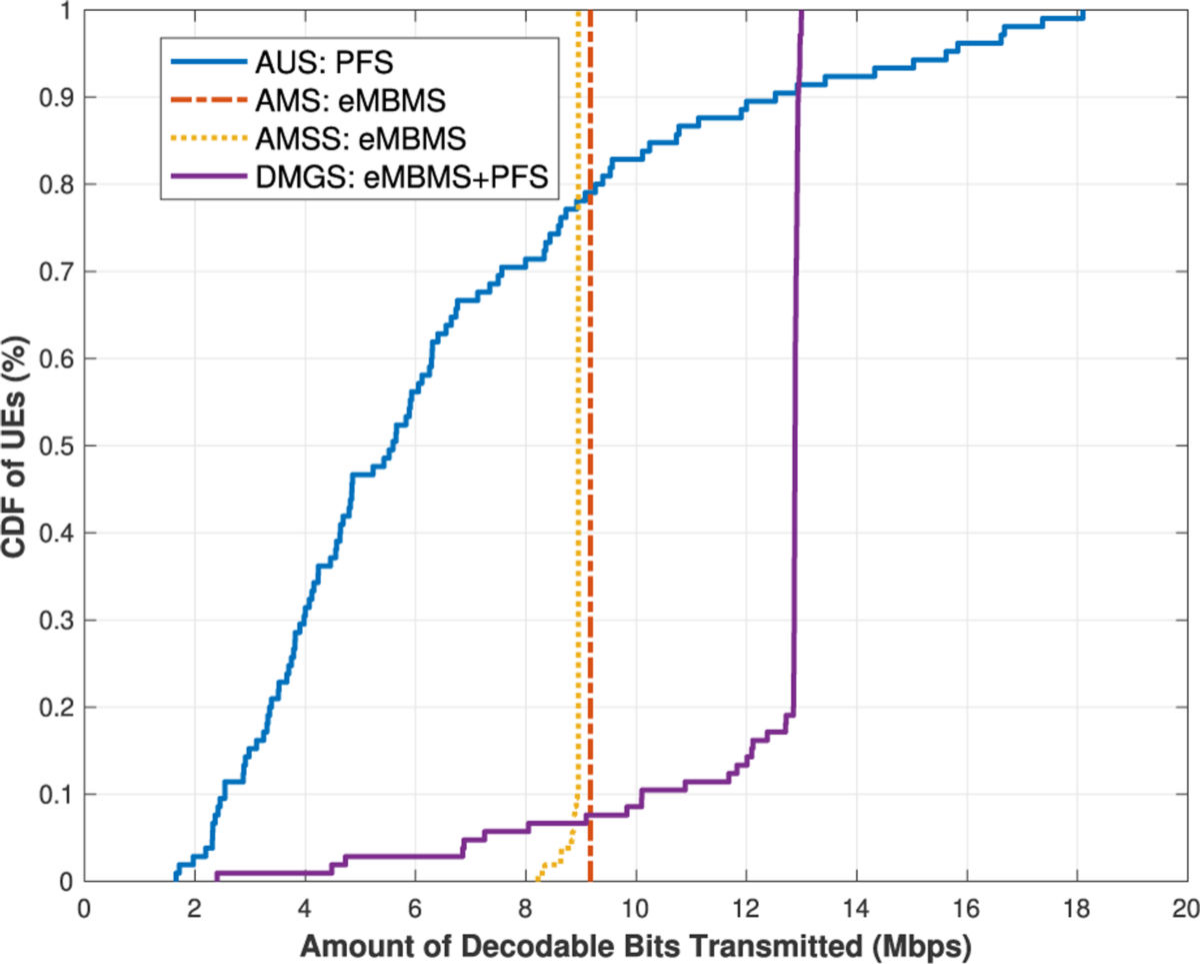
UEs’ throughput for Sc. I.a.: 5 UEs per sector.

**FIGURE 5.**
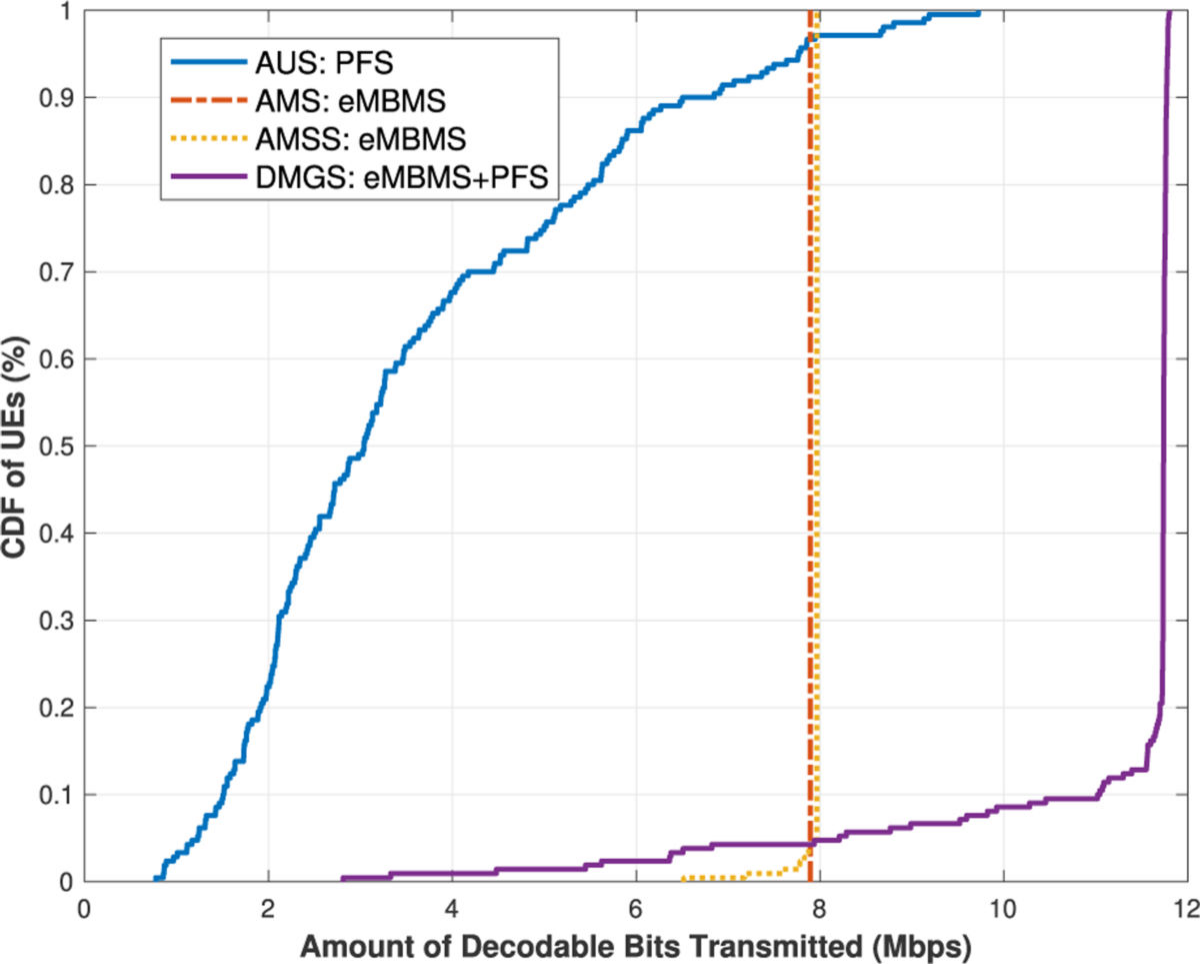
UEs’ throughput for Sc. I.b.: 10 UEs per sector.

§ First we would like to observe the network UEs’ throughput results for the algorithms under the best effort model. As it can be seen in Figure 4 and 5, the trends of the results of the **AUS** and **AMS** schemes in both cases generally align with those presented in [3]. In Table 4, we list the average UE throughput values of the four algorithms for both cases.

When comparing the results of the **AMS** and **AMSS** schemes, we would expect that **AMS** out-performs **AMSS**, due to the rational that the sampled CQI values are approximately stable within the *T*_*c*_ window, but do not always reflect the most accurate or best available CQI values at the current TTI; hence the throughput would then be impaired, as shown in Figure 4. However, this argument does not match with the results when there are 10 UEs per sector. This is because that, despite the fact that some of the UEs will not be able to decode the TB in a given TTI, the others who are capable can utilize the resources more efficiently, as shown in Figure 5, and thus the overall network performance is raised.

These results, from another perspective, also showcase that, our proposed coherence time sampling scheme is quite effective, in which the sampled values do indeed properly reflect the true channel quality values at most of the times. This type of result is also shown in our later scenarios. A separate validation experiment was conducted with large amount of data simulated with similar settings. The statistics agree with the above observation, but the details are omitted here due to limited space.

Meanwhile, in both cases, it is as expected that the results of the **DMGS** algorithm significantly out-performs all the other three baseline algorithms, especially in the second case. Note that, as we can see near the tails on the right of the **DMGS** CDFs, the curves are almost vertical lines. This is due to the fact that about 80 % of the network UEs are exclusively receiving multicast deliveries almost all the time. Conversely, in this scenario, only about 20 % of the UEs are utilizing unicast deliveries at some point due to various reasons. However, when comparing the **DMGS** with **AMS** or **AMSS**, simply because of this 20 % of UEs utilized unicast at some point, in **DMGS**, the rest of the UEs are able to utilize the channel much more efficiently, and have their throughput enhanced by up to 50 %.

Lastly, for the algorithms under the best effort model, we observe the coverage metric via the *Cell Edge User Throughput* [17] metric, i.e., the maximum throughput that covers at least 95 % of the network UEs. As listed in Table 5, for the **AUS** scheme, although some of the UEs can achieve really high data rate, the coverage performance is discouragingly low in both cases. On the contrary, it is shown that our **DMGS** algorithm is still capable of providing relatively satisfactory coverage results. If we relax the coverage to 90 % of the UEs, then the **DMGS** algorithm is capable of providing almost the same degrees of coverage without even taking into consideration the GBR factor; specifically, 10.09 Mb/s and 11.01 Mb/s (refer to the CDFs), respectively.

§ For evaluating the performance under the GBR model, we measure the average *Flight Time* metric, i.e., the average time it would take for ALL the network UEs to reach the amount of data required by the GBR. This metric will help identifying the **amount of delay** that might be introduced to the communications. For **Sc I.a.**, we set Q = 3.00 Mb/s, which is usually considered as the recommended GBR for standard definition video streaming; and given this GBR value, only 1 % of the UEs cannot reach the GBR under **AUS**. Meanwhile, for **Sc I.b.**, we set Q = 1.50 Mb/s, which is usually regarded as the minimum requirement for video streaming; and in this case, all UEs can reach the GBR. As shown in Table 6, when under the same category, the **DMGS** scheme again significantly out-performs the other schemes.

In summary, for the baseline scenarios, we showed that our proposed **DMGS** algorithm can effectively enhance the network *capacity* and *coverage*; as well as considerably reduce the end-to-end *delay* upper-bound for GBR carriers. Below, we discuss and evaluate some specially picked deployment scenarios that would more realistically reflect various PS incidents to show that our proposed algorithm is anomaly resilient. Note that, in later scenarios, without losing generality, we only observe cases where there are 10 UEs per sector.

### C. SCENARIO II. URBAN SETTINGS

In this family of scenarios, we consider a much smaller MBSFN that consists of only 3 neighboring eNB cells, namely sectors 2,6, and 7 in Figure 3, and the FR UEs are located within the area of the three adjacent sectors of these eNBs, denoted as the *Tri-Sector Area*. We consider this family of scenarios to be more realistic as it can be mapped to an urban environment setting; specifically, at a random location in the city, it is usually surrounded by several eNB cell sites/antennas hanging on nearby building walls.

In this scenario, we consider two UE deployment cases. In Sc. II.a, we consider uniformly randomly distributed UEs in the tri-sector area. Whereas in Sc. II.b., we consider that majority of the FR UEs are approximately rallied around an incident scene in the center of the tri-sector area, which is often the UE topology case in real life scenarios.

**FIGURE 6.**
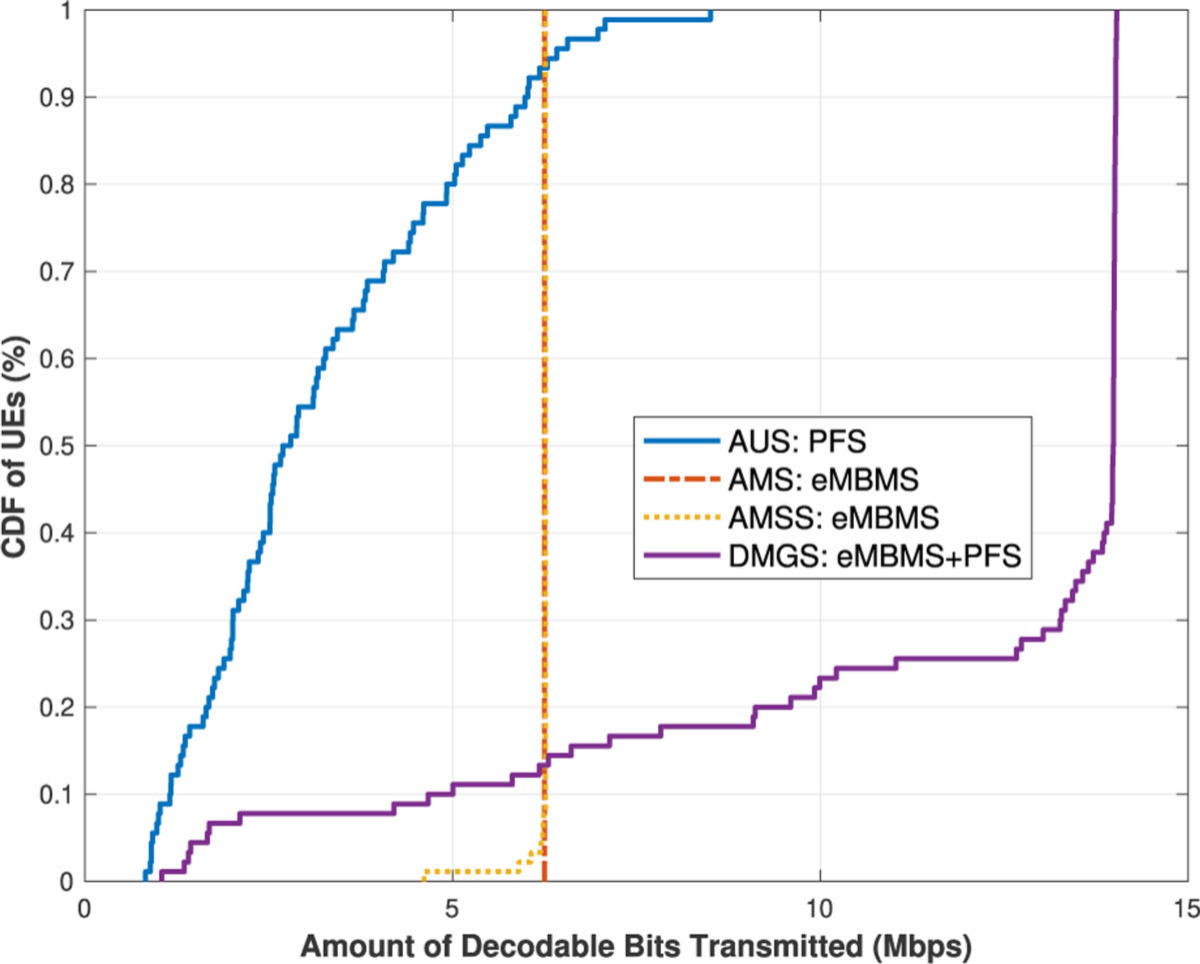
UEs’ throughput for Sc. II.a.: uniformly random UEs.

**FIGURE 7.**
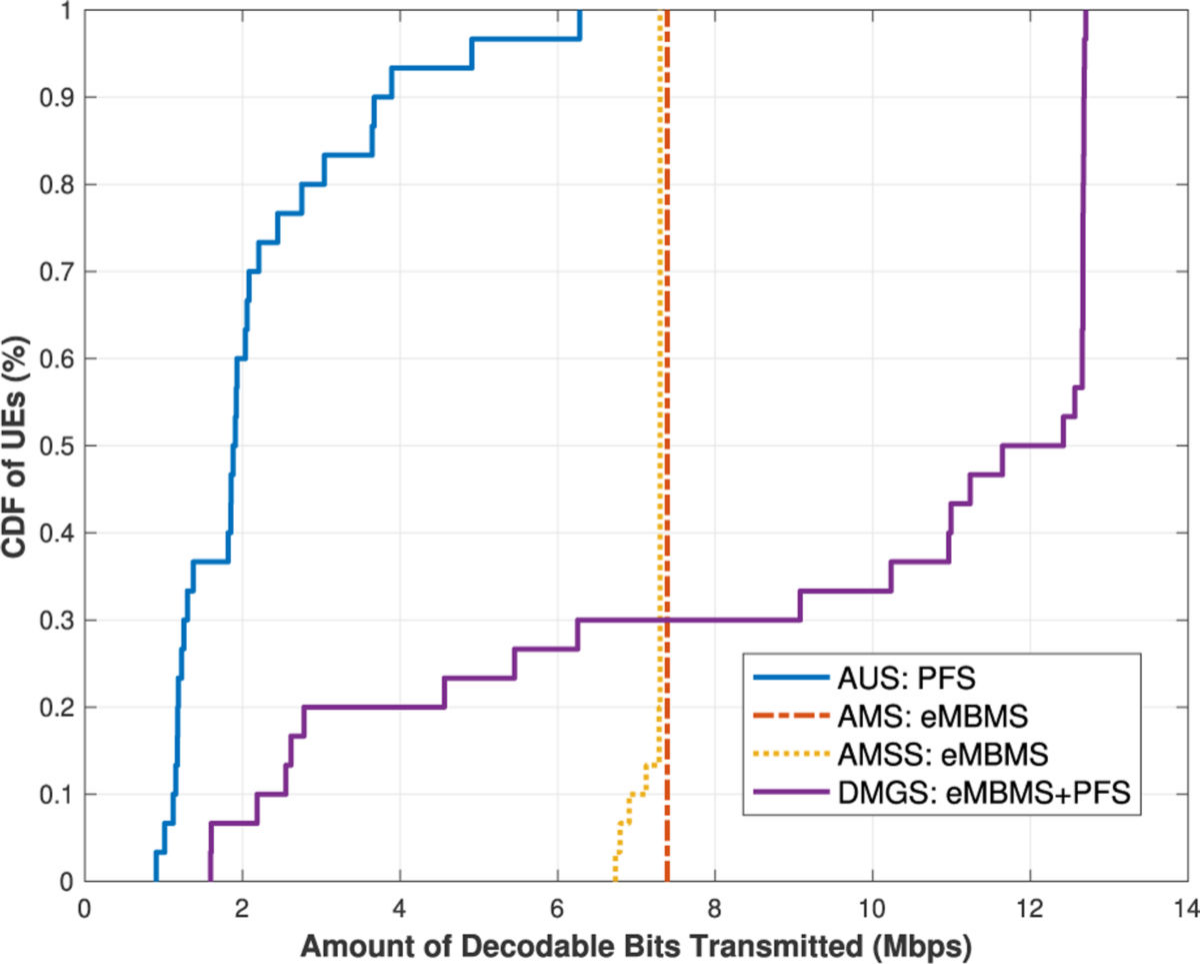
UEs’ throughput for Sc. II.b.: UEs at tri-sector center.

§ The best effort throughput results of the algorithms for the two cases are presented in Figure 6 and 7, and the average UE throughput in Table 7. As we can see, the trend of these results are largely as expected as the **DMGS** algorithm out-performs the others quite substantially. However, in this scenario, since there are fewer eNB sites (3 instead of 7) that are contributing to the constructive ICI, the MBSFN gain for the UEs are not as much as those in the previous scenario. Consequently, in **DMGS**, more UEs are going to utilize unicast deliveries at some point; specifically, now at about 40 % to 55 %. This not only means that more UEs are now utilizing their MIMO capabilities, which is what we want to achieve through our proposed solution, but also that it helps the rest of the UEs to further increase their multicast performance, since they can now utilize much better multicast MCS values.

However at the same time, due to weaker MBSFN gains, the advantages of the **DMGS** over the others is in fact visibly shortened; as we can see in the average throughput metric, but especially for the coverage metric. As we can see in Table 8, now the 90 % coverage for our proposed algorithm for Sc. II.a. is less than 5 Mb/s, which is almost one third of the achievable average throughput. For Sc. II.b., the 90 % coverage is only about 2 Mb/s, which is only 1 Mb/s higher than that of the **AUS** scheme; which is not to mention the cell edge coverage metric.

§ For the GBR model, since UEs who have reached the GBR will no longer be taken into consideration in the scheduling procedure, UEs who are currently lacking will then be provided with more opportunities. Hence, for Sc. II.b., we observe a correlation between the GBR Q and the 90 % coverage performance for the **DMGS** algorithm, as shown in Table 9. It can be seen that, once we enable the GBR factor in the scheduling process, much higher levels of coverage can be easily achieved; for instance, now almost all network UEs are covered by Q = 5.00 Mb/s mark, which is regarded as the recommended GBR for high definition video streaming.

In summary, for scenarios like this, although the coverage performance is not ideal under the best effort model, when switched to the GBR model, our proposed **DMGS** algorithm can still be quite effective, and would still be able to provide a moderate coverage bit rate amount for the network UEs. These results demonstrate the resiliency of our proposed scheme.

### D. SCENARIOS III. LOCAL SETTINGS

Last but not least, in the third scenario family, we consider a single cell MBSFN scenario, where the MBSFN consists of only the three sectors of the central eNB, and the FR UEs are located within these sectors. We consider this scenario to be also quite realistic, since it can be mapped to a local incident environment, where the impacted area is confined, i.e., within the range of a single cell. In this situation, the UEs are going to experience the minimum amount of MBSFN gain, and the main advantage left for utilizing eMBMS is the ability to share the radio spectrum.

In this scenario, we also consider two UE deployment cases, utilizing a cell partitioning approach similar to the concept presented in [18]. In Sc. III.a., we consider that majority of the UEs are within a medium range from the eNB in the cell. Whereas in Sc. III.b., we consider that majority of the UEs are either close to the center of the cell or on the edge of the cell. We consider this type of cell partitioning approach to be realistic in PSNs, because different types of FRs might be rallied at different locations from a disaster scene. For example, while firefighters are on-scene of a burning building, paramedics are tending victims at further away perimeters.

**FIGURE 8.**
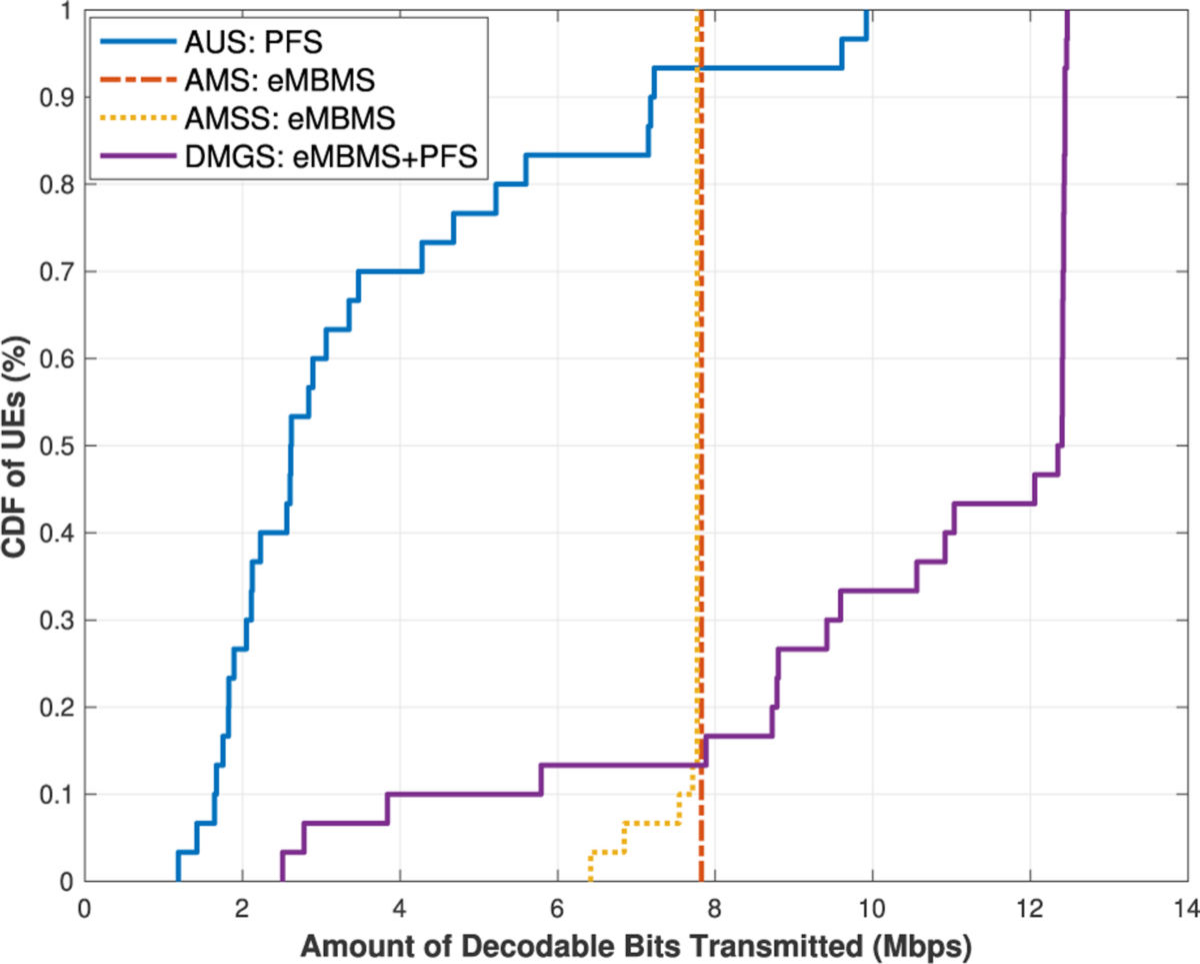
UEs’ throughput for Sc. III.a.: UEs at mid-range in cell.

**FIGURE 9.**
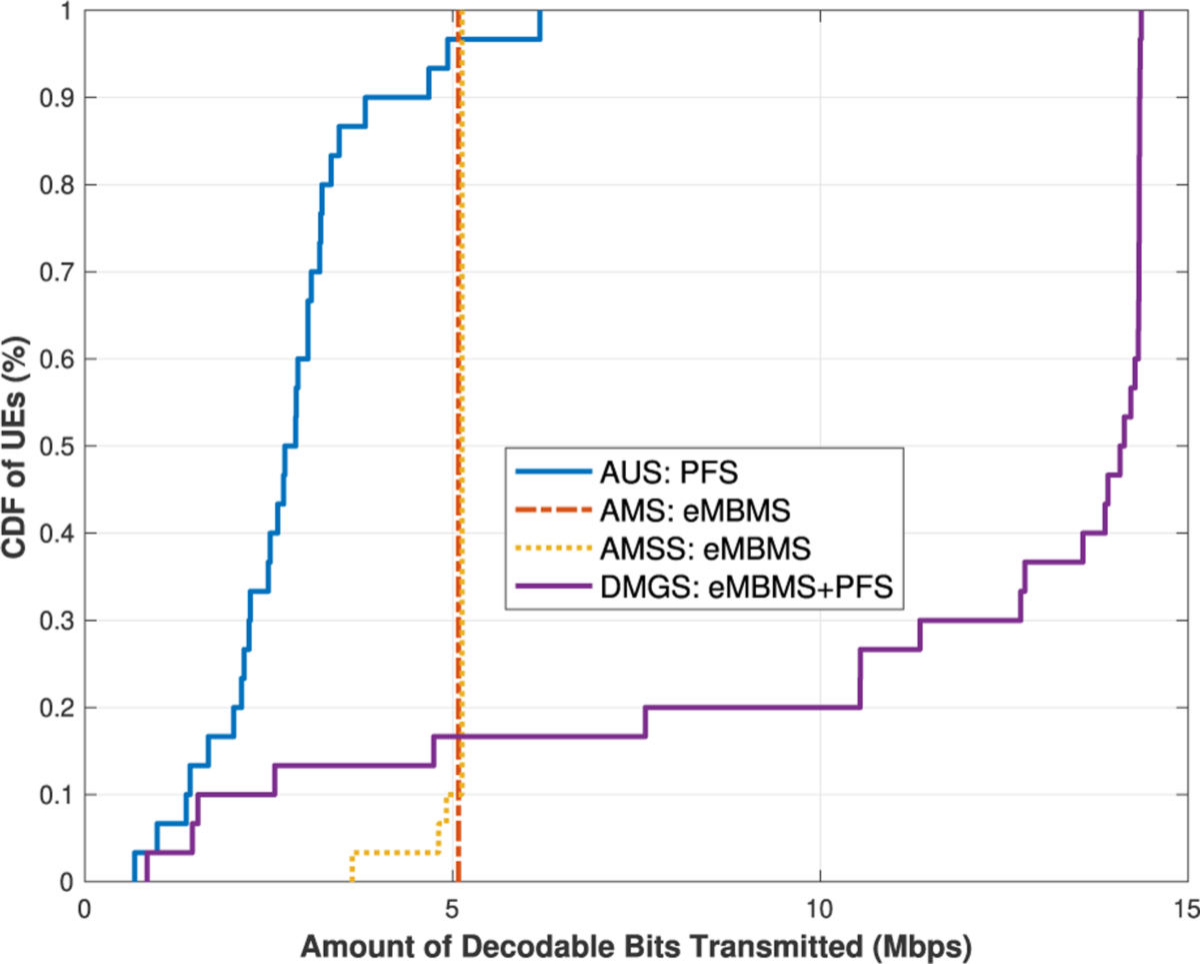
UEs’ throughput for Sc. III.b.: UEs at cell center and edge.

§ Once again, as shown in Figure 8 and 9 and Table 10, the proposed **DMGS** algorithm out-performs others quite significantly. Furthermore, due to even less MBSFN gains, even more UEs are utilizing unicast deliveries at some point during the transmissions, now 50 % to 60 %.

When comparing the two cases, as it can be seen from Table 10, that since UEs at mid ranges are likely to experience better channel qualities than those at the cell edges, and some close to the eNB, due to antenna angle, the performance for Sc. III.a. is higher than those for Sc. III.b. in the three baseline algorithms. However, as it can be seen, since **DMGS** could better utilize both types of traffic, its performance for Sc. III.b. is, conversely, better than that for Sc. III.a., even though in the latter case, UEs’ channel qualities are less ideal.

Now, since the MBSFN factor is the minimum among UEs, as we can see in Table 11, the 90 % coverage performance is almost strictly limited by UEs’ with the worst capable channel qualities, especially for the second case.

§ When considering the GBR model, we are expecting that our **DMGS** would indeed enhance the coverage performances. Our results show that, for Sc. III.a., when the GBR Q is smaller or equal to 5.50 Mb/s, we can guarantee that at least 90 % of the UEs will reach the GBR; and when Q ≤ 5.00 Mb/s, 95 % coverage can be achieved. Similarly, for Sc. III.b., when Q ≤ 4.00 Mb/s, 90 % coverage can be achieved; and when Q ≤ 3.50 Mb/s, 95 % coverage can be achieved; which, both, more than doubled the achievable value under the best effort model. Thus, these results, once again, prove that our proposed solution is resilient to the changing conditions of the environment.

### E. OTHER PERFORMANCE MEASURES

In the above analyses, we see that the proposed **DMGS** algorithm can effectively reduce the amount of *flight time*, which defines the delay upper bound for a given GBR value. Additionally, the *Queuing Delay* metric can also be studied. For the **AMS** and **AMSS** schemes, an almost constant 2 ms delay should be expected between two bursts of packets transmissions; while for **AUS** scheme, this delay will mostly depend on the number of UEs within each sector. The queuing delay for the **DMGS** will hence always lie between those of the previous cases, and, in general, tends to perform better than that of the **AUS**.

*Fairness* of the scheduling is another key metric; yet, for our across the board solution, defining what is “fair” would worth a full study by itself, which we will look into in the future. While, intuitively, since **DMGS** exhibits much lower coefficient of variance levels in the achieved throughput values when compared with those of the **AUS** scheme in all the studied scenarios, **DMGS** also tends to have a higher fairness level among all the scheduled UEs.

## VI. CONCLUSION AND FUTURE WORK

In this work, we developed an effective and efficient dynamic eMBMS Multicast grouping scheduling algorithm in MBSFNs for public safety scenarios. The **DMGS** algorithm leverages the advantages of both multicast and unicast traffic; and is shown to be capable of significantly enhancing the mission critical *capacity* and *coverage* performances for the deployed FRs. We exhibited the resiliency of our proposed solution by showing that it is capable and suitable for providing and sustaining high performance services for vastly different types of public safety incidents. Our consistent results further prove that eMBMS multicast is a prominent solution in overcoming limitations in near future PSNs.

However, the use case for eMBMS and MBSFN are not limited to PSNs; for example, in resolving the bottleneck in broadcasting popular sport/entertainment events with rigorous high-definition and low-latency requirements to massive crowds across large areas. This also makes eMBMS multicast extremely prominent for near future commercial usages, for instance under the fifth generation (5G) New Radio (NR) settings, in which we do believe that our proposed DMGS algorithm will work even more effectively, since more slot-based transmissions can be done within one fixed sampling window and under one scheduling decision.

In this work, we aimed to guarantee the service deliveries for FRs demanding the same mission-critical content over other regular UEs in the network. For our next step, we aim to bring the priorities among different services aspect into our scheduling procedure; and hence allowing preemptive scheduling for mission-critical UEs demanding content with higher priorities over other mission-critical UEs whose services have lower priorities. These, when combined with the QoS-awareness provided by DMGS, will enable the QoS, Priority, and Preemption (QPP) features envisioned for PSNs, and further enhance the MCC performance for FRs.

Lastly, extensive investigation regarding the MBSFN gain performance under various MIMO configurations is also underway. Hence, further performance evaluations could be done for the DMGS scheme with respect to the results.

## Figures and Tables

**TABLE 1.** A UE’s frequency selective and time variant channel qualities.

**TTI Index** →			35	36	37	…
**Multicast TB CQIs**			0	14	13	…
**Unicast RIs**			2	3	1	…
**Unicast CQIs**	**PRB Index** ↓	50	〈7, 6〉	〈6, 6〉	〈10, −1〉	…
		…	…	…	…	…
		1	〈5, 5〉	〈5, 5〉	〈8, −1〉	…

**TABLE 2.** Channel coherence time for ITU channel mobility models.

Mobility Model	Ped-B	Veh-A	Veh-B
**MAX Speed** *v* (km/h)	10	30	120
**Coherence Time** $T_{c}^{*}$ (ms)	59.87	19.96	4.99
**Scheduling Window** *T*_*c*_ (ms)	60	20	5

**TABLE 3.** Simulated UE deployment scenarios and their corresponding parameter settings.

UE Scenario	Description	MBSFN Sectors	UE Sectors	Number of UEs	UE Distributions
**Sc. I.a.**	Baseline	1 to 21	1 to 21	5 per sector	Uniformly Randomly Distributed
**Sc. I.b.**	Baseline	1 to 21	1 to 21	10 per sector	Uniformly Randomly Distributed
**Sc. II.a.**	Urban Baseline	1 to 9	2,6,7	10 per sector	Uniformly Randomly Distributed
**Sc. II.b.**	Urban Incident	1 to 9	2,6,7	10 per sector	Majority at Tri-Sector Center
**Sc. III.a.**	Local Incident	1,2,3	1,2,3	10 per sector	Majority at Mid-Range in Cell
**Sc. III.b.**	Local Incident	1,2,3	1,2,3	10 per sector	Majority at Cell Center and Edge

**TABLE 4.** Comparisons of UEs’ best effort throughput performance.

Algorithm	Avg. Throughput: Sc. I.a.	Avg. Throughput: Sc. I.b.
**AUS**	6.56 Mb/s	3.57 Mb/s
**AMS**	9.17 Mb/s	7.89 Mb/s
**AMSS**	8.92 Mb/s	7.94 Mb/s
**DMGS**	12.23 Mb/s	11.31 Mb/s

**TABLE 5.** Comparisons of UEs’ best effort cell edge coverage performance.

Algorithm	Cell Edge Coverage: Sc. I.a.	Cell Edge Coverage: Sc. I.b.
**AUS**	2.31 Mb/s	1.16 Mb/s
**AMS**	9.17 Mb/s	7.89 Mb/s
**AMSS**	8.82 Mb/s	7.93 Mb/s
**DMGS**	6.87 Mb/s	7.93 Mb/s

**TABLE 6.** Comparisons of average flight time for required amount of data.

Algorithm	Avg. Flight Time: Sc. I.a., 3.00 Mb	Avg. Flight Time: Sc. I.b., 1.50 Mb
**AUS**	480 ms	426 ms
**AMS**	342 ms	209 ms
**AMSS**	335 ms	200 ms
**DMGS**	255 ms	129 ms

**TABLE 7.** Comparisons of UEs’ best effort throughput performance.

Algorithm	Avg. Throughput: Sc. II.a.	Avg. Throughput: Sc. II.b.
**AUS**	3.22 Mb/s	2.16 Mb/s
**AMS**	6.24 Mb/s	7.38 Mb/s
**AMSS**	6.23 Mb/s	7.24 Mb/s
**DMGS**	11.81 Mb/s	9.44 Mb/s

**TABLE 8.** Comparisons of UEs’ best effort cell edge coverage performance.

Algorithm	90 % Coverage: Sc. II.a.	90 % Coverage: Sc. II.b.
**AUS**	1.15 Mb/s	1.11 Mb/s
**AMS**	6.24 Mb/s	7.38 Mb/s
**AMSS**	6.26 Mb/s	6.90 Mb/s
**DMGS**	4.66 Mb/s	2.18 Mb/s

**TABLE 9.** Comparisons of average flight time for required amount of data.

**GBR Q (Mb/s)**	4.50	5.00	5.50	6.00
**90 % Coverage (Mb/s)**	4.50	4.84	4.62	4.59
**Avg. Flight Time (ms)**	448	463	476	447

**TABLE 10.** Comparisons of UEs’ best effort throughput performance.

Algorithm	Avg. Throughput: Sc. III.a.	Avg. Throughput: Sc. III.b.
**AUS**	3.61 Mb/s	2.79 Mb/s
**AMS**	7.82 Mb/s	5.08 Mb/s
**AMSS**	7.68 Mb/s	5.05 Mb/s
**DMGS**	10.38 Mb/s	11.56 Mb/s

**TABLE 11.** Comparisons of UEs’ best effort cell edge coverage performance.

Algorithm	90 % Coverage: Sc. III.a.	90 % Coverage: Sc. III.b.
**AUS**	1.64 Mb/s	1.37 Mb/s
**AMS**	7.82 Mb/s	5.08 Mb/s
**AMSS**	7.54 Mb/s	4.91 Mb/s
**DMGS**	3.83 Mb/s	1.53 Mb/s

## Footnotes

1 **Disclaimer**: The identi_cation of any commercial product or trade name does not imply endorsement or recommendation by the National Institute of Standards and Technology, nor is it intended to imply that the materials or equipment identi_ed are necessarily the best available for the purpose.

2 Unlike the Future-eMBMS (FeMBMS) de_ned in Rel. 14, where almost all the resources are available for multicast, but only in secondary cells.
